# Geoelectrical resistivity data sets for subsurface characterisation and aquifer delineation in Iyesi, southwestern Nigeria

**DOI:** 10.1016/j.dib.2017.10.057

**Published:** 2017-10-26

**Authors:** Ahzegbobor P. Aizebeokhai, Kehinde D. Oyeyemi, Funmilola R. Noiki, Blessing I. Etete, Akpore U.E. Arere, Ubongabasi J. Eyo, Valentino C. Ogbuehi

**Affiliations:** Applied Geophysics Group, College of Science and Technology, Covenant University, Ota, Nigeria

**Keywords:** Geoelectrical resistivity, VES and 2D ERI, Subsurface characterisation, Aquifer delineation, Groundwater potential

## Abstract

This article consists of geoelectrical resistivity data sets for thirty (30) vertical electrical sounding (VES) and four (4) traverses of 2D electrical resistivity imaging (ERI) collected within Iyesi, Ota, southwestern Nigeria for about five (5) weeks between December, 2016 and January, 2017 using an ABEM Terrameter (SAS1000/4000). The observed apparent resistivity data sets for the VES were processed using WinResist to obtain geoelectric layer parameters while those of the 2D ERI were processed with RES2DINV to obtain 2D inverse model resistivity images. The geoelectric parameters for the VES and the inverse models for the 2D ERI were integrated to characterise the subsurface and delineate the underlying aquifer units.

**Specifications Table**

**Table:** 

Subject area	Geophysics
More specific subject area	Geoelectrical Resistivity
Type of data	Table, Text file, DAT, RST, and INV files
How data was acquired	Geoelectrical Resistivity Survey using ABEM Terrameter (SAS1000/4000)
Data format	Raw, Processed
Experimental factors	The observed apparent resistivity data sets were processed for subsurface characterization and aquifer delineation.
Experimental features	Geoelectrical resistivity survey for VES and 2D ERI was conducted.
Data source location	Iyesi is between latitude 6°39′−6°41′ and longitude 3°09′−3°11′ in the eastern Dahomey basin, southwestern Nigeria.
Data accessibility	All the data sets are with this article.

**Value of the data**

• The datasets can be used for geoelectrical resistivity characterization of the subsurface and the lithologic units within the depth of investigation can be delineated.
• The datasets can be used for groundwater potential studies.
• The depth and thickness of the aquifer unit within the study area can be determined and used for siting boreholes and wells in groundwater development.
• The data can be integrated with other geophysical data sets such as electromagnetic, ground penetrating radar gravity and seismic data for detail subsurface characterization.
• The data set can be used for educational purposes, and for future research in hydrogeological, environmental and geotechnical studies.
• The data can be compared with those obtained from similar geologic environment.

## 1 Data

The attached files (Appendices A and B) contain vertical electrical soundings (VES) and 2D electrical resistivity imaging (ERI) data sets for subsurface characterisation and aquifer delineation. The raw data sets are presented for both VES and 2D ERI in ‘dot DAT’ format (DAT files); the processed VES data sets are presented in ‘dot RST’ format while those of 2D ERI are presented in ‘dot INV’ format.

## 2 Experimental design, materials and methods

### 2.1 Study area

The study area is Iyesi, Ota, Ogun State, southwestern Nigeria; it is between latitude 6°39′−6°41′ and longitude 3°09′−3°11′. The topography is gentle sloping and elevation averaged about 75 m above mean sea level. The regional climate is tropical humid characterized by two distinct seasons – dry and rainy seasons. Precipitation is generally heavy rainfall which distinguishes the climatic seasons. Annual mean rainfall is greater than 2300 mm and forms the main sources of groundwater recharge in the area; monthly temperature ranges from 23 °C in July to 32 °C in February.

The study area is located within the eastern Dahomey basin, an extensive basin that stretches along the continental margin of the Gulf of Guinea from southern Ghana through Togo and Benin Republic on the west side; the basin is separated from the Niger Delta in the eastern section by the Benin Hinge Line and Okitipupa Ridge [1], [2]. Rocks in the basin are generally Late Cretaceous to Early Tertiary in age and the stratigraphy includes Abeokuta, Ewekoro, Akinbo, Oshosun, Ilaro and Benin Formations [3], [4], [5]. The local geology of the study area is predominantly coastal plain sands and Recent sediments. The coastal plain sands consist of poorly sorted clayey sands, reddish mud/mudstone, clay and sand lenses, and sandy clay with lignite of Miocene to Recent. These sediments are underlain by a sequence of coarse sandy estuarine, deltaic and continental beds and are largely characterised by rapid facies changes; thus, these sediments belong to the Ilaro Formation.

### 2.2 Data acquisition

The geophysical survey consists of VES and 2D ERI; the data sets were measured with ABEM Terrameter (SAS1000/4000) during the dry season (December, 2016 and January, 2017). A total of thirty (30) VESs were conducted using Schlumberger array with maximum half-current electrode spacing (AB/2) ranging from 180 m to 420 m. The goal of the VES survey was to delineate the subsurface lithologic layering, and determine depth-to-aquifer and aquifer geometry. The procedure for conducting VES has been discussed in several research articles e.g. [6], [7]. The GPS coordinates and surface elevation for the VESs points are presented in Table 1.

**Table 1:** GPS coordinates and elevations of the VES points.

**VES**	VES1	VES2	VES3	VES4	VES5	VES6	VES7	VES8	VES9	VES10
**Easting**	3.17987	3.17857	3.17847	3.17792	3.17612	3.17485	3.17500	3.17430	3.17357	3.17318
**Northing**	6.65692	6.65747	6.65590	6.65785	6.65982	6.65927	6.65560	6.65543	6.65540	6.66712
**Elev. (m)**	56.0	57.0	56.0	58.0	63.0	60.0	62.0	58.0	60.0	61.0
**VES**	VES11	VES12	VES13	VES14	VES15	VES16	VES17	VES18	VES19	VES20
**Easting**	3.17380	3.17482	3.17287	3.17223	3.17208	3.17203	3.16993	3.17127	3.17140	3.17230
**Northing**	6.65443	6.65483	6.65403	6.65472	6.65397	6.65340	6.65108	6.65347	6.65418	6.65540
**Elev. (m)**	60.0	58.0	59.0	53.0	53.0	52.0	51.0	51.0	55.0	60.0
**VES**	VES21	VES22	VES23	VES24	VES25	VES26	VES27	VES28	VES29	VES30
**Easting**	3.17237	3.17133	3.17075	3.17075	3.16937	3.18030	3.17982	3.17710	3.17758	3.17643
**Northing**	6.65742	6.65720	6.65888	6.65635	6.65428	6.65412	6.65335	6.65347	6.65168	6.65102
**Elev. (m)**	61.0	60.0	61.0	60.0	54.0	52.0	51.0	54.0	55.0	56.0

The survey for the 2D ERI was conducted along four (4) traverses using Wenner array; each of the traverses was 500 m in length, except for Traverse 3 which was 600 m long. The electrode separation for the data measurements ranges from 10.0 m to 160.0 m in an interval of 10.0 m (10.0 m to 200.0 m was used for Traverses 3). Survey techniques for measuring 2D ERI data can be found in the following articles [8], [9], [10]. The GPS coordinates of some of the electrode locations are presented in Table 2.

**Table 2:** GPS coordinates and elevations of the electrode positions for the 2D ERI.

		**Traverse 1**				**Traverse 2**		
**Electrode no.**	**Electrode position**	**Easting**	**Northing**		**Elevation (m)**	**Easting**	**Northing**	**Elevation (m)**
1	0.0	3.17660	6.65702		59.0	3.17238	6.65547	56.0
6	50.0	3.17697	6.65677		60.0	3.17278	6.65537	58.0
11	100.0	3.17735	6.65655		57.0	3.17335	6.65530	59.0
16	150.0	3.17773	6.65628		56.0	3.17380	6.65532	59.0
21	200.0	3.17810	6.65603		60.0	3.17422	6.65542	57.0
26	250.0	3.17848	6.65583		59.0	3.17457	6.65548	59.0
31	300.0	3.17890	6.65565		58.0	3.17498	6.65560	57.0
36	350.0	3.17935	6.65545		58.0	3.17547	6.65567	59.0
41	400.0	3.17972	6.65525		56.0	3.17592	6.65560	59.0
46	450.0	3.18012	6.65512		57.0	3.17639	6.65540	60.0
51	500.0	3.18055	6.65497		56.0	3.17697	6.65537	59.0
		**Traverse 3**				**Traverse 4**		
1	0.0	3.17178		6.65065	50.0	3.16932	6.65223	50.0
4	30.0	3.17170		6.65092	49.0	3.16930	6.65250	51.0
7	60.0	3.17170		6.65117	49.0	3.16933	6.65276	51.0
13	120.0	3.17173		6.65170	50.0	3.16932	6.65330	52.0
19	180.0	3.17177		6.65223	50.0	3.16933	6.65385	53.0
25	240.0	3.17183		6.65678	51.0	3.16938	6.65438	54.0
31	300.0	3.17193		6.65332	52.0	3.16960	6.65488	55.0
37	360.0	3.17207		6.65382	53.0	3.16958	6.65542	58.0
43	420.0	3.17212		6.65433	56.0	3.16940	6.65592	59.0
49	480.0	3.17220		6.65488	57.0	3.16927	6.65643	61.0
51	500.0	3.17223		6.65505	56.0	3.16920	6.65658	61.0
55	540.0	3.17230		6.65538	58.0			
61	600.0	3.17237		6.65597	60.0			

### 2.3 Data processing

The field curves for the VES data sets were curve-matched with Schlumberger master curves to estimate geoelectric layer parameters which were used as the initial models for computer iteration on a Win-Resist program to obtain model geoelectric parameters for the delineated layers. Similarly, the data sets for the 2D ERI were processed and inverted using RES2DINV inversion code, which is a non-linear optimization technique for determining 2D resistivity distribution [9], [11]. Least-squares inversion technique with standard least-squares constraint (L_2_-norm), which minimizes the square of the difference between the observed and the computed apparent resistivity, was used for the data inversion. The least-squares equation for the inversion was solved using the standard Gauss-Newton optimization technique and appropriate damping factors for the inversion were selected based on the estimated noise level on the measured data.

## Footnotes

Appendix A Supplementary data associated with this article can be found in the online version at http://dx.doi.org/10.1016/j.dib.2017.10.057.

## References

[1] Onuoha K.O. (1999). Structural features of Nigeria's coastal margin: an assessment based on age data from wells. J. Afr. Earth Sci..

[2] Wilson R.C.C., Williams C.A. (1979). Oceanic transform structures and the developments of Atlantic continental margin sedimentary basin: a review. J. Geol. Soc. Lond..

[3] Billman H.G. (1992). Offshore stratigraphy and Paleontology of Dahomey (Benin) Embayment. NAPE Bull..

[4] Okosun E.A. (1990). A review of the Cretaceous stratigraphy of the Dahomey Embayment, West Africa. Cretac. Res..

[5] Omatsola M.E., Adegoke O.S. (1981). Tectonic evolution and Cretaceous stratigraphy of the Dahomey Basin. Niger. J. Miner. Geol..

[6] Aizebeokhai A.P., Oyeyemi K.D., Joel E.S. (2016). Groundwater potential assessment in a sedimentary terrain, southwestern Nigeria. Arab. J. Geosci..

[7] Aizebeokhai A.P., Oyebanjo O.A. (2013). Application of vertical electrical soundings to characterize aquifer potential in Ota, Southwestern Nigeria. Int. J. Phys. Sci..

[8] Aizebeokhai A.P. (2010). 2D and 3D geoelectrical resistivity imaging: theory and field design. Sci. Res. Essays.

[9] Loke M.H., Barker R.D. (1996). Practical techniques for 3D resistivity surveys and data inversion. Geophys. Prospect..

[10] Loke M.H., Chambers J.E., Rucker D.F., Kuras O., Wilkinson P.B. (2013). Recent developments in the direct-current geoelectrical imaging method. J. Appl. Geophy..

[11] Griffiths D.H., Barker R.D. (1993). Two dimensional resistivity imaging and modelling in areas of complex geology. J. Appl. Geophys..

